# The distal femoral epiphysis in forensic age diagnostics: studies on the evaluation of the ossification process by means of T1- and PD/T2-weighted magnetic resonance imaging

**DOI:** 10.1007/s00414-022-02927-6

**Published:** 2022-12-24

**Authors:** Natia Chitavishvili, Ismini Papageorgiou, Ansgar Malich, Maria L. Hahnemann, Gita Mall, Hans-Joachim Mentzel, Daniel Wittschieber

**Affiliations:** 1 Section of Pediatric Radiology, Department of Radiology, Jena University Hospital, Friedrich Schiller University Jena, Jena, Germany; 2 Department of Radiology, Jena University Hospital, Jena, Germany; 3 Institute of Radiology, Südharz Klinikum Nordhausen, Nordhausen, Germany; 4 Institute of Legal Medicine, Jena University Hospital, Friedrich Schiller University Jena, Am Klinikum 1, 07747 Jena, Germany

**Keywords:** Forensic age diagnostics in the living, Ossification process, Knee joint, Age of majority, Forensic radiology

## Abstract

The age of majority, which corresponds to the age of 18 years in most European countries, plays a crucial role for a large number of legal decisions. Accordingly, an increasing number of requests by authorities to forensic age estimation experts comprise the question of whether the age of 18 years has been reached by an individual. In recent years, novel study data suggested that magnetic resonance imaging (MRI) of the knee might likewise allow for the determination of majority beyond reasonable doubt. However, the data basis, especially concerning the distal femoral epiphysis (DFE), is still poor. For this reason, 392 routine MRI cases of the knee (204 males and 188 females of a Western Caucasian population, aged between 12 and 25 years) were retrospectively analyzed. T1-weighted and water-selective fat-saturated PD/T2-weighted sequences, generated at 1.5 and 3.0 T clinical MR scanners, were available. Ossification stages of the DFE were determined by means of the classification system by Vieth et al. (Eur Radiol 2018; 28:3255–3262). Both the intra-observer agreement and inter-observer agreement were found to be “very good” (*κ* = 0.899 and *κ* = 0.830). The present study confirmed that MRI of the DFE is suitable to determine majority in both sexes when stage 6 is present as the study revealed minimum ages above the age of 18 years for this stage (20.40 years in males and 20.60 years in females). Accordingly, the data represent a strong support for the so far existing database. Hence, the investigation of the knee using routine MRI appears to become a realistic alternative for forensic age estimation practice in the near future.

## Introduction

In the recent years, expert opinions on forensic age estimations are still numerously demanded by different courts and other authorities across Europe, essentially fostered by increasing cross-border migration [1]. If such migrants do not have valid identification documents, e.g., due to insufficient official birth registration, loss of documents while fleeing, or due to the attempt of benefitting from of age-dependent financial or social resources, questions concerning the chronological age of an individual may arise in different legal situations of civil and criminal law [2]. In particular, the age of majority, which corresponds to the age of 18 years in most European countries, plays a crucial role for a large number of legal decisions to ensure the protection of the rights of minors, and to prevent the reduction of the minors’ resources by adults [3].

To determine whether the legal age threshold of 18 years has been exceeded in an individual, the Study Group on Forensic Age Diagnostics (AGFAD) of the German Society of Legal Medicine (DGRM) currently recommends the supplementary assessment of the ossification stage of the medial clavicular epiphysis [4] using projection radiography, which must actually be considered obsolete today [5], or computed tomography. However, as both methods include X-ray radiation that usually require a particular legal basis for application in forensic age estimations [3], there is a strong and increasing interest in the establishment of methods without ionizing radiation to evaluate skeletal age. In contrast to ultrasound, magnetic resonance imaging (MRI) has already intensively been investigated regarding its suitability in forensic age diagnostics [6–10].

As early as in 2015, Ottow et al. were able to show that majority can basically be determined in both sexes beyond reasonable doubt by means of MRI of the clavicles [11]. In the following years, studies with other and larger case cohorts corroborated this finding [12, 13]. In 2018, Vieth et al. [14] introduced a novel classification system for the two epiphyses of the knee joint requiring both T1- and T2-weighted MRI at 3.0 T. The study suggested that MRI of the knee might likewise allow for the determination of majority beyond reasonable doubt. Meanwhile, first validation studies at 1.5 T [15–17] and at 0.31 T (low-field MRI) [18] have been published. However, the data basis, especially concerning the distal femoral epiphysis (DFE), is still poor. Thus, the present study aims at validating the classification system by Vieth et al. [14] in the DFE. In addition, the study strives to enlarge the general database in order to facilitate the practical application of this methodology in future forensic age diagnostics.

## Materials and methods

### Assembly of the study cohort

With approval of the local ethics committee of the Jena University Hospital (reference number “2019–1362-Daten”) and the ethics committee of the medical association of the Federal State of Thuringia (reference number “53,394/2019/129”), MRI scans of one knee joint of 442 subjects between 12 and 25 years of age were collected and analyzed retrospectively. A difference between left and right side was not made. The scans were originally generated between 2010 and 2019 at the Südharz Klinikum Nordhausen (Academic Teaching Hospital of the Jena University Hospital) and at the Institute of Diagnostic and Interventional Radiology of the Jena University Hospital. The main indication for these scans was the assessment of a possible internal knee trauma. The medical records of the subjects did not contain any disease affecting skeletal growth or trauma or post-surgical lesions of the knee. During the case collection, an effort was made that age and sex distribution of the cases are as even as possible. The same study cohort was already used in a previous study investigating the ossification process of the proximal tibial epiphysis by means of MRI [17].

Image quality concerning the distal femoral epiphysis (DFE) was assessed using a Likert scale from 1 to 3 (1 = poor, 2 = good, 3 = excellent quality). During the later evaluation, 50 cases had to be excluded due to poor image quality (Likert 1, *n* = 23), or due to different artifacts impeding the determination of the ossification stage (*n* = 27), e.g., movement artifacts or superimpositions by bone marrow edema.

The final study cohort comprised 392 assessable knee MRI scans (204 males and 188 females) (Table 1). Due to the names and places of birth within the medical records of the subjects as well as due to the general population structure of the Federal State of Thuringia located in Central Germany, the vast majority of these cases can be regarded as part of a Western Caucasian population. Hence, a high socio-economic status of the study cohort can be assumed.

**Table 1:** Number of assessable cases by age and sex (*n* = 392)

Age group (years)	Females	Males
12	15	13
13	16	18
14	12	15
15	15	12
16	12	15
17	12	15
18	11	15
19	14	15
20	16	14
21	13	12
22	16	17
23	16	15
24	11	17
25	9	11
Σ	188	204

### Imaging parameters

Because of the retrospective study design, no additional protocols were applied. The MRI scans of the study were performed according to standard procedures at various MR scanners at 1.5 T or 3.0 T (Siemens Healthineers, Erlangen, Germany; Philips, Eindhoven, The Netherlands). The ossification stage of the DFE was determined using coronal views only. Each case evaluation was performed by means of two different MR sequences as described below (in brackets: first, the data for 1.5 T, and after slash, the data for 3.0 T):

- A T1-weighted turbo spin echo sequence (T1w TSE; TR 540/750 ms; TE 7.3/19 ms; flip angle 90/120°; field of view 160 mm; slice thickness 3.0 mm) and
- A proton-density-weighted turbo spin echo sequence with fat suppression (PD TSE FS) (TR 3390/4720 ms; TE 30/39 ms; flip angle 90°; field of view 160 mm; slice thickness 3.0 mm) or
- A PD/T2-weighted turbo-inversion recovery-magnitude sequence (TIRM) (TR 3720/3770 ms; TE 32/80 ms; flip angle 173/143°; field of view 160 mm; slice thickness 3.0 mm).

### Image analysis

**Fig. 1:**
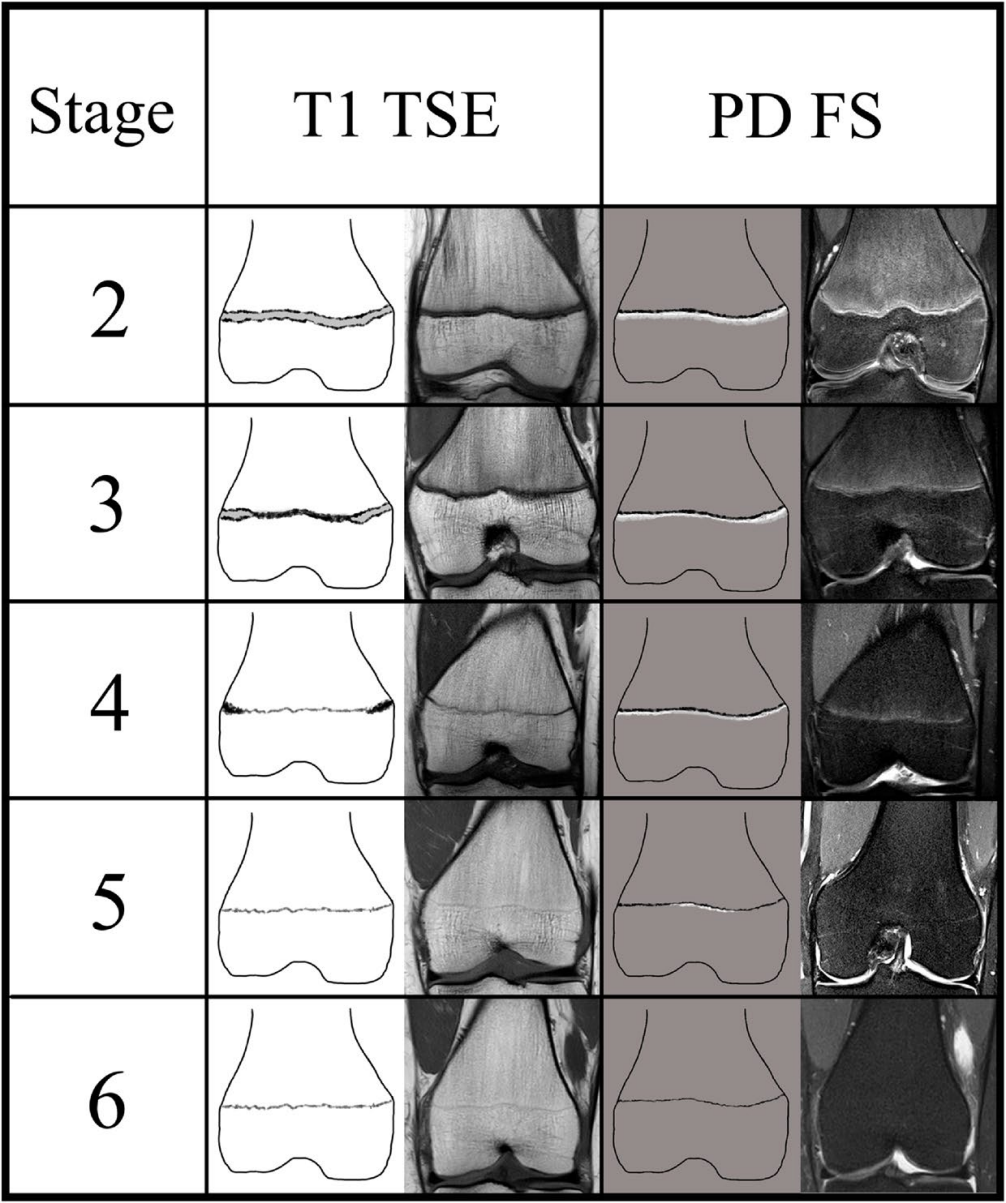
The classification system by Vieth et al. [14] applied to the DFE. Schematic drawings and case examples from our study cohort

All MRI scans were evaluated at a standard PACS workstation and certified monitors. The degree of the ossification of the DFE was assessed by means of the classification system by Vieth et al. [14] (Table 2, Fig. 1). During all image assessments, identity, age, and sex of the individuals were always unknown for the readers.

**Table 2:** Original descriptions of the ossification stages defined by Vieth et al. [14]. Bold text highlights relevant differences between the stages

Stage	Type of sequence	Original descriptions
Stage 2	T1	A continuous band of intermediate signal intensity is visible, walled by serrated lines of low to no signal intensity towards the epiphysis and the diaphysis
	T2	The epiphysis is demarked by a serrated line of low to no signal intensity. The metaphysis shows two serrated lines of high signal intensity. Both lines can be continuous or discontinuous
Stage 3	T1	A discontinuous band of intermediate signal intensity is visible. The band is walled by serrated lines of low to **no signal intensity towards the epiphysis and the diaphysis that sporadically convene and interrupt the band, forming a single serrated line with no signal intensity**
	T2	The metaphysis shows two serrated lines of **high signal intensity that sporadically convene, forming a single thin and serrated line of high signal intensity**
Stage 4	T1	A discontinuous thin and serrated line of intermediate signal intensity between the epiphysis and the diaphysis is visible. In the continuity of the line, **thicker sections with no signal intensity** can be seen
	T2	A thin single, discontinuous or dotted line of hyperintense signal is visible in the same position as the described thin line of the corresponding T1-w sequence. In the continuity of the line, **thicker hyperintense sections** can be seen
Stage 5	T1	A continuous thin line of intermediate signal intensity between the epiphysis and the diaphysis is visible
	T2	A single thin, discontinuous or dotted line of **hyperintense signal** in the same position as the described thin line of the corresponding T1-w sequence
Stage 6	T1	A continuous thin line of intermediate signal intensity between the epiphysis and the diaphysis is visible
	T2	**No hyperintense signal** in the same position as the described thin line of the corresponding T1-w sequence

For the final statistical parameters, three readers performed the assessments consensually: Reader 1 was a pediatric radiologist, and reader 2 was a forensic physician, each of which with more than 10 years of specific experience in skeletal imaging for the purpose of forensic age diagnostics. Reader 3 was a radiological trainee and doctoral candidate in pediatric radiology.

Two months after the consensual assessment, 100 cases (26%) were randomly chosen to assess intra- and inter-observer agreement. Then, ossification stage determinations were done again by readers 1 and 3, and again, 2 months after the first re-assessment by reader 3 alone.

### Statistical analysis

All statistical analyses were done using IBM SPSS Statistics Version 27 (release 17 June 2020). The statistical parameters for the ossification stages were expressed as minimum, maximum, mean ± standard deviation, and median with lower and upper quartiles. Sex-related differences were tested by means of the Mann–Whitney *U* test for two independent groups. The effect of the image quality on stage determination (determined by Likert scale) was calculated using Spearman’s rank correlation (r_s_). *P* < 0.05 (exact, two-sided) was considered statistically significant.

Cohen’s kappa (κ) non-parametric test was used for the evaluation of intra- and inter-observer agreements. The system proposed by Altman was employed for interpreting κ values [19]: *κ* < 0.20, poor agreement; *κ* = 0.21–0.40, fair agreement; *κ* = 0.41–0.60, moderate agreement; *κ* = 0.61–0.80, good agreement; *κ* = 0.81–1.00, very good agreement.

## Results

The MR image quality concerning the DFE, which was assessed on a Likert scale from 1 to 3, did not reveal a significant effect on the process of stage determination (Spearman’s correlation coefficient r_s_ =  − 0.027, *p* = 0.782, and repetition after 2 months with the same patients, Spearman’s correlation coefficient r_s_ = 0.025, *p* = 0.798).

**Fig. 2:**
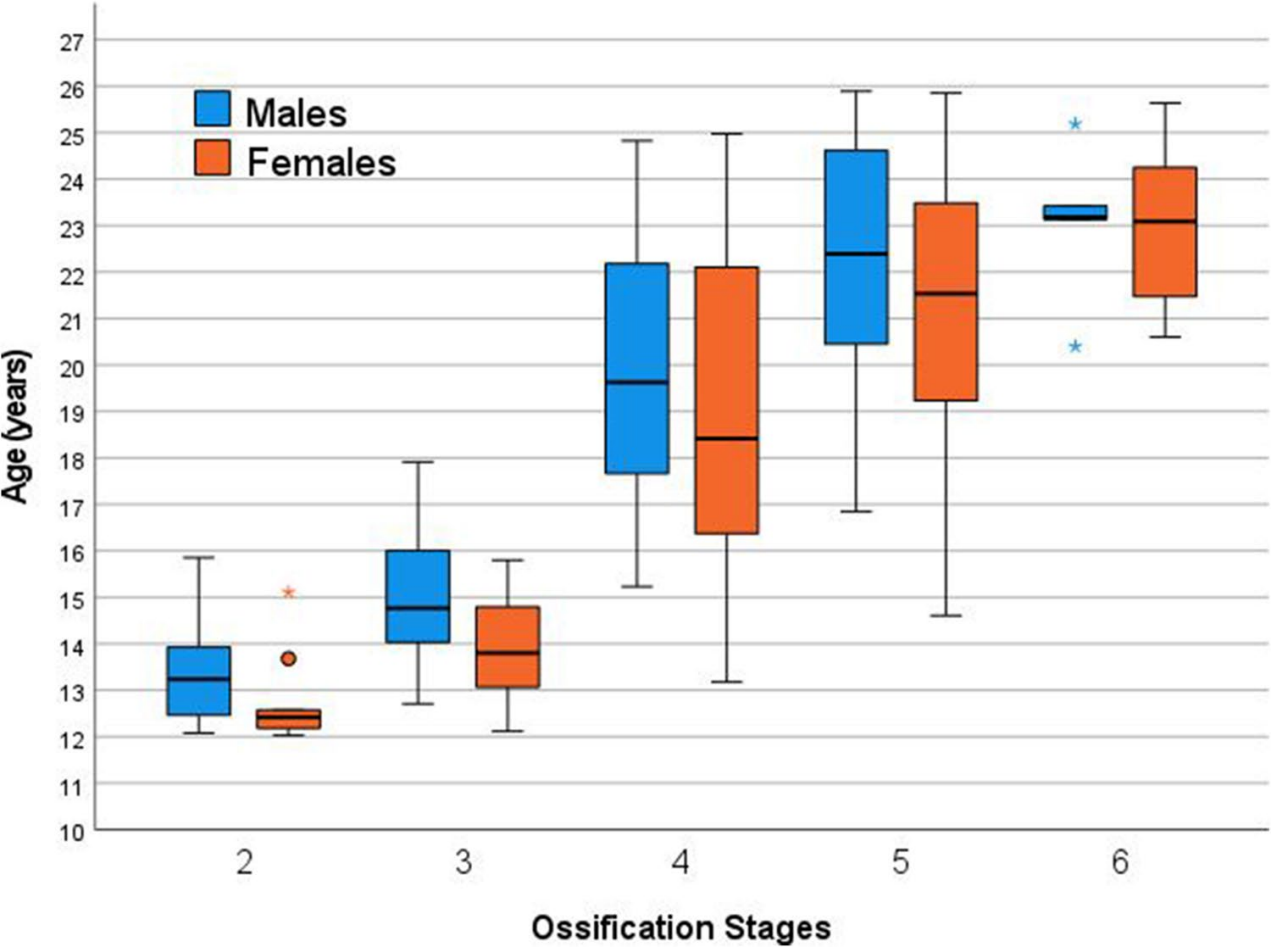
Box-and-whisker plot diagram showing the age ranges obtained for all five ossification stages (stages 2–6) and for both sexes. Whiskers indicate the minimum and maximum ages, unless otherwise indicated by stars (statistical outliers)

The statistical parameters of the final stage determinations are presented in Table 3 and additionally visualized by means of a box-and-whisker plot diagram in Fig. 2. Age medians are constantly increasing from stage to stage for both sexes, confirming a good discrimination of the ossification stages of the applied classification system. An accelerated development can be assumed for male individuals. A statistically significant sex-related difference was found for stage 3 only (*p* = 0.001). Moreover, a relatively large scatter from the age minimum to the age maximum is striking for stages 4 and 5 in both sexes. Stage 6 was determined infrequently only (*n* = 12). In males, the stages 2, 3, 4, 5, and 6 were first observed at the ages of 12.08, 12.71, 15.23, 16.85, and 20.40 years. In females, the stages 2, 3, 4, 5, and 6 were first observed at the ages of 12.03, 12.11, 13.18, 14.61, and 20.60 years.

**Table 3:** Synopsis of the statistical parameters expressed in years (*n* = 392). Note that in both sexes, the minimum ages of the stages 2 and 3 as well as the maximum ages of the stages 5 and 6 must not be used in age estimation practice due to truncation of the age interval of the study population. *Minimum*, *minimum age*; *Maximum*, *maximum age*; *SD*, *standard deviation*; *LQ*, *lower quartile*; *M*, *median*; *UQ*, *upper quartile*; *statistically significant

Stage	Sex	*n*	Minimum	Maximum	Mean ± SD	LQ │ M │ UQ	*p* (male vs. female)
2	Male	28	12.08	15.85	13.33 ± 1.03	12.46 │ 13.24 │ 13.93	0.064
	Female	10	12.03	15.11	12.73 ± 0.96	12.17 │ 12.42 │ 12.85	
3	Male	38	12.71	17.92	14.98 ± 1.34	13.95 │ 14.77 │ 16.03	0.001*
	Female	32	12.11	15.80	13.93 ± 1.02	13.06 │ 13.80 │ 14.81	
4	Male	68	15.23	24.83	20.06 ± 2.68	17.60 │ 19.63 │ 22.21	0.060
	Female	69	13.18	24.98	19.12 ± 3.28	16.31 │ 18.41 │ 22.14	
5	Male	65	16.85	25.89	22.20 ± 2.47	20.42 │ 22.39 │ 24.66	0.069
	Female	70	14.61	25.86	21.27 ± 2.78	19.22 │ 21.53 │ 23.49	
6	Male	5	20.40	25.19	23.07 ± 1.71	21.77 │ 23.18 │ 24.31	1.000
	Female	7	20.60	25.64	22.97 ± 1.95	21.15 │ 23.09 │ 25.26

Intra- and inter-observer agreements were calculated by means of κ statistics to assess repeatability and reproducibility of the staging method applied. As to the comparison of the 100 stage determinations by reader 3 (first re-assessment) with those by reader 3 (second re-assessment), the intra-observer agreement corresponded to a “very good agreement” (*κ* = 0.899). Regarding the comparison of the 100 stage determinations by reader 1 with those by reader 3, the inter-observer agreement was found to be a “very good agreement” as well (*κ* = 0.830).

## Discussion

As recently demonstrated for the proximal tibial epiphysis [17], the present study on the ossification process of the distal femoral epiphysis (DFE) again corroborated the applicability of the classification scheme by Vieth et al. [14] for routine MRI data at 1.5 T and 3.0 T. Although the T2-TSE SPIR sequence of the original study was not available, the information required for applying the staging system could be replaced by other water-selective sequences with fat saturation, which were used in the present study. These results are also in line with data obtained by two Turkish studies [15, 16] investigating MRI scans of the knee by means of the classification system by Vieth et al. [14]. Table 4 shows a comparison of the four currently available studies using MRI of the DFE and presenting statistical parameters obtained by application of the Vieth stages.

**Table 4:** Comparison of the studies investigating MRI of the DFE using the classification system by Vieth et al. [18]. *HDI*, *human development index 2021/2022 *[20]; *T1w*, *T1 weighted*; *T2w*, *T2 weighted*; *TSE*, *turbo spin echo*; *SPIR*, *spectral pre-saturation with inversion recovery*; *FS*, *fat suppressed*; *PD*, *proton density*; *SPAIR*, *spectral attenuated inversion recovery*; *TIRM*, *turbo inversion recovery magnitude*

	Vieth et al. (2018) [14]	Gurses et al. (2020) [15]	Alatas et al. (2021) [16]	Present study
General study characteristics				
Number of cases	694	598	709	392
Study design	prospective	retrospective	retrospective	retrospective
Age groups [years]	12–24	12–30	12–27	12–25
Geographic origin of the study cohort	Germany	Turkey	Turkey	Germany
HDI rank of the country	9	48	48	9
Technical parameters				
Field strength(s)	3.0 T	1.5 T	1.5 T	1.5 T / 3.0 T
T1w sequence	TSE (coronal)	TSE (sagittal/coronal)	TSE (coronal)	TSE (coronal)
Slice thickness (T1w)	3.0 mm	3.5 mm	3.5 mm	3.0 mm
T2w sequence(s)	TSE SPIR (coronal)	FS PD TSE (coronal)	PD SPAIR TSE (coronal)	PD TSE FS or TIRM (coronal)
Slice thickness (T2w)	3.0 mm	3.0 mm	3.5 mm	3.0 mm
Minimum ages of the ossification stages [years]				
Stage 2 (males)	12.05	12.08	12.02	12.08
Stage 2 (females)	12.11	12.08	12.01	12.03
Stage 3 (males)	12.13	12.92	12.34	12.71
Stage 3 (females)	12.16	12.92	12.01	12.11
Stage 4 (males)	15.49	14.33	14.84	15.23
Stage 4 (females)	14.33	15.08	13.77	13.18
Stage 5 (males)	15.71	14.75	15.81	16.85
Stage 5 (females)	14.82	15.83	14.77	14.61
Stage 6 (males)	21.24	20.58	20.76	20.40
Stage 6 (females)	20.65	20.58	20.45	20.60

Consistent with all previous studies, the present study confirmed that MRI of the DFE is suitable to determine majority (age of 18 years) in both sexes when stage 6 according to Vieth et al. [14] has been determined. The minimum ages of stage 6 were 20.40 years in males and 20.60 years in females, which is nearly perfectly in line with the two previous studies from Turkey [15, 16]. However, Vieth et al. [14] found the presence of stage 6 in males as late as at age 21.24 years, and therefore occurring at first above the legal age threshold of 21 years. Due to this discrepancy, we re-assessed the 20-year-old male individual with stage 6 and, however, had to confirm the previous evaluation as definitely no hyperintense signal, defining stage 5, could be detected. Thus, on the one hand, our data support the assumption that the presence of stage 6 in males does not indicate the completion of the age of 21 years. On the other hand, it has to be considered that PD-weighted sequences are less sensitive towards watery components than T2-weighted sequences, which might possibly explain the present discrepancy.

With respect to stage 6, it was striking that this stage was observed in 12 cases only (3.0% of the total study cohort), which is at least similar to the original study by Vieth et al. (12/694 = 1.7% [14]), but markedly different to the two previous Turkish studies (165/598 = 27.6% [15], 70/709 = 9.9% [16]). As it appears unlikely that Turkish study populations generally include proportionally more stage 6 cases, and as populations with a probably lower socio-economic status would rather have been expected a deceleration of the ossification process with a lower case number of the final stage 6, we hypothesize a difference in the process of stage determinations.

Regarding stages 4 and 5, a relatively large scatter from age minimum to age maximum was found in both sexes. Compared to the data by Vieth et al. [14], the present study revealed strikingly many “stage 4 cases” above the initially reported age maxima (18.81 years in males, 18.46 years in females [14]), which actually suggests a systematic bias in determining stage 4. However, re-evaluation of these “stage 4 cases” revealed that, in our opinion, these cases are correctly determined as stage 4. On that occasion, it was noticeable that, in many of these cases, the features of stage 4, such as “thicker sections with no signal intensity” (corresponding to unfused physeal growth plate), were detectable as comparably small phenomena, especially at the very posterior parts, or at the very lateral and/or medial parts of the femoral condyles, and might therefore quickly be overlooked. It might be promising to investigate whether the stage 4 can be sub-divided into sub-stages (4a, 4b, and 4c) in order to make further age diagnostic statements, e.g., concerning the ages of 16 and 18 years.

As also reported previously [14, 17], we were able again to draw the relevant landmarks of each stage from the T1-weighted sequence; except for the important stage 6, which requires information from an additional water-selective sequence with fat saturation: a faint hyperintense signal at the physeal growth plate. Unlike with our experience in the previous study on the proximal tibial epiphysis [17], the differentiation between stages 5 and 6 was not a problem in the present study on the DFE. However, it has to be considered that a considerably lower number of stage 6 cases was observed in the DFE cohort. On the whole, different ossification stages in T1 and PD TSE FS/TIRM occurred very rarely. Merging of the information of both sequence types was done according to modified rules originally developed for CT slices of the medial clavicular epiphysis [21, 22]: stage 2 (in one sequence) + stage 3 (in the other sequence) → stage 3, stage 3 + stage 4 → stage 4, stage 4 + stage 5 → stage 4, and differentiation between stages 5 and 6 only possible by PD TSE FS/TIRM [14].

Inherently, the present study also contains several limitations. The retrospective study design definitely represents the most relevant limitation. Although the study approach comprised a 10-year period and the database of two tertiary care hospitals, only 392 cases could be included in the final study cohort, mainly due to strict inclusion criteria. This case number is markedly lower than the 694 cases of the first study by Vieth et al. [14]. Nevertheless, we believe that the data of the present study may serve as a valuable support for the so far existing database.

Concerning the noteworthy strengths of the study, it can be stressed that our study group already had previous experiences [17] with the relatively novel classification system by Vieth et al. [14], thereby warranting an increased level of reliability regarding the stage determinations. In addition, contrary to the above-mentioned Turkish studies [15, 16], where a lack of information on the socio-economic status of the study subjects was stated, the socio-economic status of the present Western Caucasian study population can be regarded as high. As also described by Vieth et al. [14], this is relevant for the practice of forensic age estimations (e.g., as to the “minimum age concept” [3]): Applying the minimum ages of a reference study, that used a study population with high socio-economic status, will rather lead to an underestimation of age when applied to individuals with a lower socio-economic status, which then usually represents no legal disadvantage. Furthermore, the two Turkish studies [15, 16] did not have an even distribution of their study subjects across sexes and age groups, which — in turn — is another strength of the present study.

Several other research groups have also studied the possibilities of the MRI technology for the purpose of forensic age estimation by means of the DFE. Dedouit et al. [23] defined 5 original MRI stages and investigated both knee epiphyses in 290 patients aged between 10 and 30 years. Although this staging system was apparently able to determine majority in case of a stage 5 [23, 24], critics complained that the approach is mainly based on the absolute measurable thickness of the growth plate layer(s), which, however, are affected by the individual’s body height and therefore actually unsuitable for absolute measurements [14]. Krämer et al. [25] investigated MRI scans of the DFE in 290 patients aged between 10 and 30 years by means of a combination of the classification systems proposed by Schmeling et al. [26] and Kellinghaus et al. [27]. The authors found a minimum age for stage 4 at 18.3 years in males. However, larger subsequent studies were not able to confirm this result [28–30], so that this approach finally turned out as not suitable for being a sole indicator of majority.

Then, since its first introduction in 2018, the classification system by Vieth et al. [14] has repeatedly been shown to be suitable for determining majority in both sexes when applied to the DFE [14–16]. Furthermore, the applicability of this classification system has also been proven for other ossification centers, such as the proximal humeral epiphysis [31], the two wrist epiphyses (distal radius and ulna) [32], and the proximal tibial epiphysis [14–17].

Thus, especially the two epiphyses of the knee joint might play a bigger role in the near future because MRI of the knee has several advantages over the so far existing alternative — MRI of the medial clavicular epiphysis: First, MRI of the knee can be performed using dedicated extremity MR scanner (eMRI), whereas MRI of the clavicles requires a conventional whole body MR scanner (wbMRI). As the image acquisition times are considerably lower in eMRI, the necessary level of cooperation is lower as well. Moreover, eMRI can also be used in patients with claustrophobia. Furthermore, it is much easier to immobilize the knee in an eMRI, whereas motion artifacts may affect the assessment of the clavicles in the wbMRI. In addition, breathing and pulsation artifacts have a considerable effect on the image quality and thereby on the validity of the examination of the clavicles. Secondly, in contrast to the medial clavicular epiphysis, anatomical shape variants do not occur in the ossification centers of the knee joint.

## Conclusions

1. The data of the present study reinforced the finding that majority can be determined in both sexes when the DFE shows a stage 6 of the classification system by Vieth et al. [14]. Accordingly, the data represent a strong support for the so far existing database.
2. All morphological features required for stage determination in the DFE were detectable in both at 1.5 and 3.0 T, even without the T2-TSE SPIR sequence used in the original study [14]. However, water-selective sequences *with fat saturation* are indispensable for stage determination.
3. Considering the growing body of recent data on both knee epiphyses in combination with the classification system by Vieth et al. [14–18], the investigation of the knee using routine MRI appears to become a realistic alternative for forensic age estimation practice in near future; either as a general additional tool or if CT of the medial clavicular epiphysis fails, e.g., when anatomical shape variants are detected on both sides. It remains to be seen whether “MRI of the knee” will be considered suitable for a possible update of the AGFAD recommendations.
4. A continuous enlargement of the database is still needed.
